# Impact of Organizational Brand-Building Strategies on Organizational Brand Equity: A Moderating Role of Brand-Oriented Leadership

**DOI:** 10.3389/fpsyg.2022.919054

**Published:** 2022-07-01

**Authors:** Zhang Wei

**Affiliations:** Department of Education, Tianjin Vocational Institute, Tianjin, China

**Keywords:** organizational brand building strategies, employee-based brand equity, organizational brand equity, brand-oriented leadership, signaling theory, theory of motivation

## Abstract

In this era of competition, branding is an essential marketing tool for organizations to compete in today's dynamic markets. Organizations should realize the importance of employee-based brand equity from the perspectives of customer branding and financial performance. Employee-based brand equity plays a crucial role in building organizational brand equity. This study conceptualized a model that helps the practitioners to build employee-based brand equity and organizational brand equity. This study examines the role of organizational brand-building strategies and brand-oriented leadership in promoting employee-based brand equity and organizational brand equity. This study collected data from the employees of various beverage companies in China. This study analyzed data through partial least square structural equation modeling using Smart PLS 3. This study found a positive direct association between organizational brand-building strategies and employee-based brand equity. However, according to the results, no direct association was found between organizational brand-building strategies and organizational brand equity. This study also confirms that organizational brand-building strategies indirectly promote organizational brand equity through employee-based brand equity. Moreover, this study demonstrates that brand-oriented leadership directly influences employee-based brand equity and organizational brand equity but negatively moderates the relationship between organizational brand-building strategies and employee-based brand equity. No moderation was found in the relationship between organizational brand-building strategies and organizational brand equity. Finally, the practical and theoretical implications of this study are discussed.

## Introduction

Branding is one of the powerful tools for organizations to compete in today's competitive market (Hasni et al., 2018). Organizations use branding to create a competitive advantage and to be more sustainable in the market (Shocker and Aaker, 1993). The concept of branding was mostly associated with products and services in the preceding decades, but now literature admits the importance of branding from the perspective of human resources. This shift of thoughts illuminated the important role of human capital (such as employees) in the successful branding of the organization (King and Grace, 2010). In this era of competition, organizations are now recognizing the importance of the intellectual abilities of employees. The importance of tangible assets for the successful branding of organizations cannot be denied, but they have to admit the significance of human capital (Boukis and Christodoulides, 2020).

The efforts put by organizations for the creation of branding assist the firms in achieving their goals of establishing brand equity (Fernández-Ruano et al., 2022). Further, they give arguments and say that higher brand equity is an encouraging indication of the sustainability of organizations. Branding could be used from a multi-dimensional perspective by organizations; however, it is considered internal branding when practiced among employees (Hasni et al., 2018). Internal branding is a precursor for employee-based brand equity (EBBE) because employees have a critical role in transforming brand capacities from organizations to customers. Huang and Sarigöllü (2014) argue that an organization's brand equity positively influences when employees efficiently transform the promise of the organization toward the customer. Further, they point out that role of employees in the creation of brand equity is very crucial as organizations are always making efforts to deliver promises accurately. However, scholars noticed that EBBE is ignored in the literature despite its importance in the organization's long-term success (Silverthorne, 2004; King and Grace, 2010; Erkmen, 2018; Boukis and Christodoulides, 2020). King and Grace (2009) also point out that literature is enriched with financial- and consumer-based brand equity, but there is a dearth in the literature on brand equity from employees' perspectives.

Organizations have to divert their attention toward value addition with employees' perspectives to be more competitive in the market (Erkmen, 2018). This study attempts to recognize the importance of EBBE for organizations. This paper is an effort to strengthen the literature on EBBE by shedding light on some important aspects which can play an important role in the creation process of EBBE. Organizations have to put considerable effort into creating brand equity from the perspective of employees (Foroudi, 2020). Firm brand-building strategies are beneficial tools for improving organizational branding. Organizational brand-building strategies are the long-term planning tools for creating brand equity. Vallaster and Lindgreen (2011) illuminated the importance of brand-building strategies and said that appropriately established approaches of organization could filter the attitude and behavior of employees about branding. Further, the efforts invested by organizations in these types of strategies motivate employees to deliver to the customers according to the promise of the organizations.

Organizational brand-building strategies act as drivers for creating organizational brand equity (Lin and Siu, 2020). It is a positive signal of organizations toward their employees when they engage them in the process of designing brand-building strategies (Lashley, 1999). When organizations take input from their employees, they indicate that their employees have worth to them. These types of initiatives by the organization could serve as determinants for the creation of EBBE. When organizations set some goals and strategies for brand building, they are trying to influence brand equity positively. The strategies build by organizations are standards for employees as by following them, they can assist the organization in the creation of brand equity. Vallaster and Lindgreen (2011) acknowledged that the role of the leader is also a considerable aspect of the brand-building process. The frontline employees have a considerable amount of effort into creating brand equity. Further, Quaratino and Mazzei (2018) added in vein and said that leaders act as the custodian of other employees in providing committed service on behalf of the organization. In addition, the leader can play a vital role in the creation process of organizational brand equity.

This study contributes to the literature on brand equity in seven ways. First, this study serves the literature on brand equity by highlighting the importance of brand equity for the sustainability of organizations. Second, this study adds some insight to the literature on organizational brand equity strategies. Based on the theory of motivation (Maslow, 1943), this study proposes that organizational brand equity strategies positively influence the employees and motivate them to create brand equity in organizations. These strategies are a positive signal for the creation of EBBE in the organizations. Third, based on signaling theory (Spence, 1973), this study assumes that organizational brand equity strategies are a positive signal of organizations for brand equity creation. Fourth, this study attempts to check the role of brand-oriented leadership in creating brand equity in an organization. Fifth, this study aims to find out the role of brand-oriented leadership in the building process of EBBE. The role of the leader is very critical in the overall brand management of the organization. That is why this study attempts to check the specific role of leaders in creating EBBE. Sixth, this study's findings also have some essential managerial implications. Seventh, this study serves the literature on brand equity by providing an empirical investigation of the building process of brand equity in organizations.

In addition, this study offers a framework for a better understanding of the brand equity creation process. For this purpose, this study proposed four different direct relationships and also mediation and moderation effects. The direct relationship of organizational brand-building strategies proposed with employee-based brand equity and organizational brand equity. The direct relationships of employee-based brand equity and brand-oriented leadership are also proposed with employee-based brand equity and organizational brand equity, respectively. The moderating role of brand-oriented leadership and the mediating role of employee-based brand equity are also considered in the proposed framework.

The remaining part of the present study is designed as follows: first, introduce the key constructs of the theoretical framework and review the literature for hypothesis development. Next, the methodology part of the paper is presented and the findings are discussed. Finally, this article is concluded with a discussion of the findings, future research directions, and limitations of the study.

## Review of Literature

### Organizational Brand Equity

Brands have been an important part of organizations' marketing strategies for hundreds of years (Feldwick, 1996). In addition, organizations placed their efforts and resources in different aspects of branding to maintain their sustainability because they realize the importance of branding to create brand equity. Shocker and Aaker (1993) define brand equity as a value-added activity of an organization to strengthen the relationship with stakeholders. Further, they acknowledged that this value addition activity could be considered from the perspective of product, trade, or customer. Feldwick (1996) shed further light on the concept of brand equity and said that organizations could measure their brand equities by gauging increments in cash flows from the perspective of the product. Further, the author elaborated on brand equity and said that this concept could be used in three different aspects. First, the concept of brand equity could be used as the value of a brand which is separate from other tangible and intangible assets. Second, brand equity could also be considered as the level of attachment that customers have with the brand. Third, the concept of brand equity could also be seen as the collection of thoughts and views of external stakeholders about the brand. Singh and Banerjee (2021) acknowledged that these three aspects are not independent as brand equity of organizations at all levels has an association with each other.

Prados-Peña and Del Barrio-García (2021) illuminated the importance of brand equity and considered it a valuable organizational asset in the literature. Further, they argue that “brand credibility and brand attitude” are two main antecedents of brand equity. Brand credibility is the level of honesty and transparency of a brand toward consumers (Singh and Banerjee, 2021). Further, they pointed out the significance of brand credibility and acknowledged that it is an important factor in long-term relationships between organizations and consumers. In addition, Prados-Peña and Del Barrio-García (2021) shed light on the importance of brand attitude and said that it could play a role as a driver for the creation process of organizational brand equity.

Aaker (1992) documented the importance of organizational brand equity and said that it is the value creation asset of an organization that can assist the firm in maintenance of sustainability in the market. Further, Faircloth et al. (2001) argue that brand loyalty, brand awareness, and brand association are three important determinants of brand equity. Brand loyalty reflects when organizations engage customers through brand equity activities because they are trying to attain the loyalty of consumers (Shocker and Aaker, 1993). Brand awareness is also a consequence of brand equity activities of an organization as it reflects the recognition of consumers about the brand (Aaker, 1992). Faircloth et al. (2001) noticed that brand association is a sense of belonging of consumers with the brand. Further, they stated that brand association is also a very important determinant of brand equity and organization put their efforts into creating brand awareness by doing branding activities.

In this era of competition, organizations are keen to find out ways to cope with the dynamics of the market. The above-discussed literature on organizational-based brand equity sheds further light on the importance of brand equity for organizations. This study aims to extend the literature by providing an empirical investigation of organizational brand equity.

### Employee-Based Brand Equity

Brand equity could be labeled as the fruitful consequence of brand-building activities of organizations (Berry, 2000). King and Grace (2010) highlighted the three important approaches to measure brand equity, including financial, customer, and employee-based brand equities. Wang (2010) defined the financial-based brand equity and said that it is the additional worth offered by a brand to a firm's economic value in the form of cash flows. According to Meng and Bari (2019) point of view, consumer-based brand equity is termed as the perceptions and feelings of consumers about the brand. In other words, this approach depicts how consumers think and behave about the brand. King and Grace (2009) shed further light on brand equity and define the EBBE as the brand value addition activity which takes place as a result of employees' efforts.

King and Grace (2010) noticed that the literature on brand equity generally considered the financial and customer approaches for measuring brand equity and ignores the third important perspective which is EBBE. However, in this era of competition, organizations have to consider the worth of EBBE-building activities (Wilden et al., 2006). Employees are an organization's assets because they have a considerable role brand-building activities of the organization (Poulis and Wisker, 2016). In addition, firms need the assistance of employees to deliver the promised value to customers. The role of human capital cannot be ignored in the brand equity creation process, as Erkmen (2018) revealed that the organization considered customer contact employees as the organization's internal customers. Brand knowledge of employees could act as a precursor for the creation process of EBBE (King and Grace, 2010). Further, Erkmen (2018) quantified employee brand knowledge as the foundation of the brand equity building process. In other words, employees with strong brand knowledge could perform more appropriately in delivering the expected brand promises.

Boukis and Christodoulides (2020) call attention to an important aspect of the brand equity building process and say that efficient knowledge dissemination is a valuable indication of brand equity cultivation. Therefore, it is the prime responsibility of the organization to give special attention to internal communication about the brand (Erkmen, 2018). In addition, effective internal communication efforts of an organization can pave the way for employees to feel psychological attachments to the organization. Scholars noticed that psychologically attached employees are more likely to engage in delivering organizational promised value to consumers (Poulis and Wisker, 2016; Fergnani, 2019). King and Grace (2010) acknowledged that employees' psychological attachment is a constructive sign toward the creation of EBBE.

King and So (2015) identified three important dimensions of EBBE including “brand-consistent behaviors, brand endorsement, and brand allegiance.” Poulis and Wisker (2016) give arguments about these three dimensions and said that brand-consistent behavior is the extent of employees by which they behave in organizational identical ways. Further, they argue that brand endorsement is the employee's positive words of mouth about the brand while brand allegiance is the intention of the employee to remain a part of the organization for the long term.

### Organizational Brand-Building Strategies

The ultimate goal of any organization is to build long-term relationships with internal and external stakeholders (Alreck and Settle, 1999). In this regard, organizations put their efforts into communicating the values of the brand to the creation of a competitive edge. However, Vallaster and Lindgreen (2011) revealed organization managers, employees, and consumers as three important actors in the brand-building process. Alreck and Settle (1999) further stated that organizations need an effective plan for brand-building activities because the comprehensive internal network is the base for successful organizational branding. King and Grace (2006) quantify these types of branding planning activities as organizational brand-building strategies. Further, they pointed out the importance of organizational brand-building strategies and said that they are playing the role of the “heart” of organizations in the equity creation process. In addition, it is stated that improved service quality, satisfied consumers, and brand loyalty could be some possible blessings in return for these activities (Quaratino and Mazzei, 2018). Scholars consider the organizational strategies as the most important component to frame the behaviors of employees in the brand-building process (Alreck and Settle, 1999; King and Grace, 2006; Vallaster and Lindgreen, 2011).

Quaratino and Mazzei (2018) also highlighted the significance of organizational brand-building strategies and said that effective managerial practices are the first step in constructing the brand-consistent behaviors of employees. Foroudi (2020) acknowledged that organizations nowadays are trying to create a competitive advantage by utilizing corporate branding internationally. According to King and Grace (2009) point of view, four important aspects could be the base of successful organizational brand-building strategies including “information generation, knowledge dissemination, openness, and the 'H' factor.” Further, they stated that information generated by conducting research enables organizations to assess crucial information about the needs and wants of employees' branding strategies. Knowledge dissemination allows organizations to transmit a framework to employees to create brand consisting behaviors (Boukis and Christodoulides, 2020). Openness indicates two-way responsibility (between organization and employee) to create a climate that is considered to be positive for the brand-building process (King and Grace, 2009). In addition, the 'H' factor is concerned with the organization's responsibility to treat employees with respect and honor as the employees have an essential part in the brand-building process.

### Brand-Oriented Leadership

The frontline employees always have a critical role in building an organizational brand image (Quaratino and Mazzei, 2018). They can put their efforts into building a positive perception of customers about the brand because these employees have direct interactions with customers. In this regard, Morhart et al. (2009) stated frontline employees as brand ambassadors who transform the organization's vision into reality. In addition, Terglav et al. (2016) point out that managers must ensure employees' commitment level for providing accurate service. For gaining expected outcomes, internal branding is a valuable effort to create brand equity. Terglav et al. (2016) point out that internal communication, training, meeting, and briefing are four important aspects of internal branding. Further, internal communication strengthens the psychological contracts between employees and organizations. The positive development of a psychological contract between both parties paves the way for reciprocity-based employment relationships (Bashir et al., 2021).

Muenjohn and Armstrong (2008) argue about the critical role of brand-oriented leadership and state that the performance of employees is highly correlated with the perception of employees about their leadership. Terglav et al. (2016) define brand-oriented leadership as the style in which they act as a role model to motivate employees to perform favorably in brand-building activities. In addition, brand-oriented leadership could draw a clear picture of brand cues in the mind of employees which in turn causes in the form of organizational expected outcomes. Successful leaders constantly and repeatedly express the importance of brand commitment and motivate employees to maintain brand promise.

Morhart et al. (2009) point out that the understanding of the creation process of brand equity could not be complete without seeking the importance of brand-oriented leadership. Boukis and Christodoulides (2020) also acknowledged that leaders have a great part in building pro-organization behaviors of employees which in turn assist the organization in creating EBBE. Further, Muenjohn and Armstrong (2008) argued about brand-oriented leadership and said that transformational leadership could play a valuable role in aligning the employee's directions toward brand-building activities. Transformational leaders always put effort into inspiring the employees and making positive changes to achieve the organizational goals (Terglav et al., 2016). However, according to Morhart et al. (2009) point of view, the transactional leadership approach can also play a considerable role in the brand-building process. Furthermore, they give arguments in favor of this approach and said that transactional leadership could align the behavior of employees toward organizational brand equity. Lee et al. (2019) also commented on the importance of transformational leadership and transactional leadership approaches and said that both have a significant role in forming the psychological process of employees and can differently influence them to engage in brand-building efforts.

### Hypotheses Development

**Figure 1:**
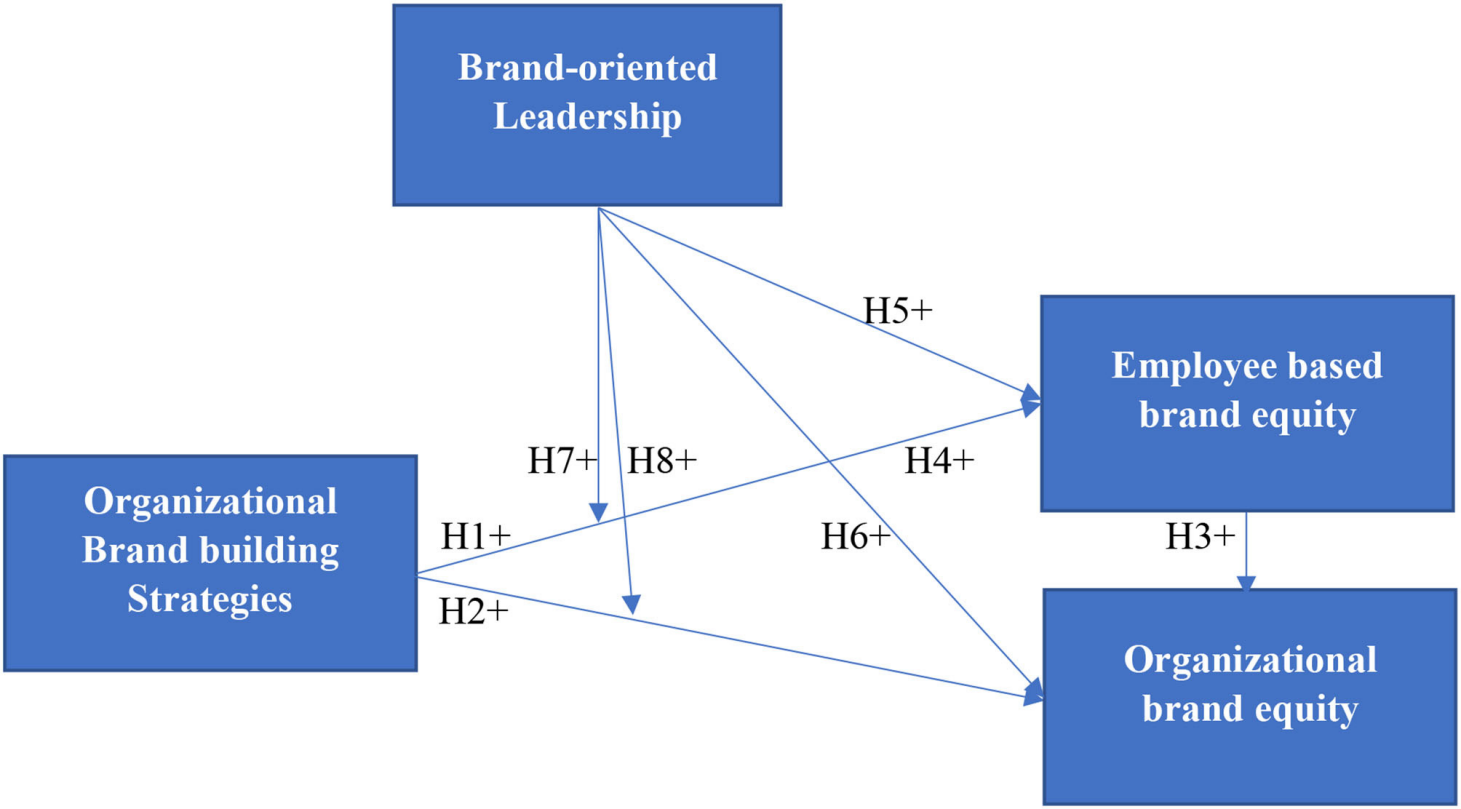
Conceptual framework.

Based on the literature, this study proposed a theoretical framework to frame the role of organizational brand-building strategies in the formation of organizational brand equity and EBBE. With the support of signaling theory, this study assumes that the organizational brand-building strategies are a positive signal for the firm to create organizational brand equity. Prados-Peña and Del Barrio-García (2021) also point out the importance of organizational brand-building strategies for brand equity building. Based on the theory of motivation, this study proposes that organizational brand-building strategies motivate employees to act in firm expected ways to create EBBE. Organizational strategies for brand equity creation always come with a fruitful outcome in creating brand equity from the employees' perspectives (King and Grace, 2009). This study also tries to determine the role of brand-oriented leadership in creating EBBE and organizational brand equity. Boukis and Christodoulides (2020) revealed that frontline employees could assist the organizations favorably in building brand equity from the perspective of employees and organizations. Further, this study also attempts to check the moderating effect of brand-oriented leadership in the creation process of organizational brand equity and EBBE. This study also tries to investigate the mediating role of EBBE in the building of organizational brand-building strategy and organizational brand equity in brand equity building. For empirical investigation, this study developed the following hypotheses. Moreover, a conceptual framework based on the above literature is represented in Figure 1.

**H1:** Organizational brand building strategies positively influence employee-based brand equity.

**H2:** Organizational brand building strategies positively influence the organizational brand equity.

**H3:** Employee-based brand equity positively influences organizational brand equity.

**H4:** Employee-based brand equity mediates the positive relationship between organizational brand building strategy and organizational brand equity.

**H5:** Brand-oriented leadership positively influences employee-based brand equity.

**H6:** Brand-oriented leadership positively influences organizational brand equity.

**H7:** Brand-oriented leadership moderates the relationship between organizational brand-building strategies and employee-based brand equity.

**H8:** Brand-oriented leadership moderates the relationship between organizational brand-building strategies and organizational brand equity.

## Research Methods

### Study Design

This study collected data under a convenient sampling technique from the employees of different beverage companies in China. The author targeted companies that are famous among people as a brand. For this purpose, the author interviewed 50 MBA executive students. The author contacted the managers of the targeted companies and explained the purpose of being contacted. Most of the managers showed their consent, and the author personally visited their offices and met with them. At the author's request, managers created a targeted employee group on the WeChat application.

The questionnaires also included a cover letter in which employees were assured that their data would be kept anonymous and used only for academic purposes, and aggregated results would be revealed. This cover letter confident the employees so that employees freely filled out questionnaires.

The author followed previous researchers' guidance for gathering suitable sample sizes. According to their guidance, up to 400 sample sizes are considered appropriate (Krejcie and Morgan, 1970; MacCallum et al., 1999). So author fixed a benchmark of 400 questionnaires. The author distributed all the questionnaires in WeChat groups. This study applied a time lag data approach to reduce common method bias. Hence, all the questionnaires were developed based on a key question for participants' response identification. Thus, this study collected data in three waves with one-month gap in each wave. In the first wave, questionnaires related to IV (Organizational brand-building strategies) and demographics were distributed among 1,200 employees.

Out of 1,200, 789 respondents filled the questionnaires. After a one-month gap, the questionnaires related to DVs (Employee-based brand equity and organization brand equity) were distributed among these 789 employees. Out of 789, 565 respondents filled the questionnaire in the second wave. Moreover, after a further one-month gap, the author distributed moderator variable (brand-oriented leadership) questionnaires among 565 respondents. Out of 565, 488 respondents completed questionnaires. Based on the key questions, the author identified the same respondents in all turns and finalized questionnaires that were complete in all respect. In this way, the author found 488 complete questionnaires.

### Measures

Participants' responses were measured through five-point Likert scale ranging from 1 (strongly disagree) to 5 (strongly agree). This study measured variables from previously used items. Organizational brand strategies were measured with the five-item scales. The five items were adapted from a previous study by Coleman, Chernatony, and Christodoulides (Coleman et al., 2011). A sample item includes “Our organization responds to our clients' brand identification needs.” The employee-based brand equity variable was measured by five items adapted from Baumgarth and Schmidt (2010), also validated by Boukis and Christodoulides (2020). A sample item includes “I always consider the impact on the company's brand when I make decisions.” Organizational brand equity was measured with seven items adapted from Srivastava (2009). A sample item includes “Does it offer advantages over the existing competitive products.” The brand-oriented leadership was measured with five items scale adapted from Boukis and Christodoulides (2020). A sample item includes “My line manager behaves consistently with the brand values, even when he is not controlled for doing so.”

### Demographics Information

Table 1 explains the demographic information of the respondents. Out of 488 participants, 276 were male, and 212 were female. A total of 112 participants were up to 28 years old, 192 participants were up to 36 years, 119 participants were up to 45 years old, and 65 participants were up to 46 or more than 46 years old. A total of 43 participants have education matric to intermediate, 126 participants have bachelor's education, 161 participants have master's education, and 158 have technical education. A total of 75 participants have <3 years of experience, 126 participants have experience of more than 3 and up to 7 years, and 161 responded have 8–10 years of experience. Ninety-six participants have 11–15 years of experience, and 30 participants have more than 15 years of experience.

**Table 1:** Demographic information.

**Categories**	**Subcategories**	**Numbers**	**Percentage**
Gender	Male	276	56.6
	Female	212	43.4
Age	20–28 years	112	23.0
	29–36 years	192	39.3
	37–45 years	119	24.4
	46 years or above	65	13.3
Education	Matric to Intermediate	43	8.8
	Bachelor	126	25.8
	Master	161	33.0
	Technical	158	32.4
Experience	<3 years	75	15.4
	4–7 years	126	25.8
	8–10 years	161	33.0
	11–15 years	96	19.7
	16 years onward	30	6.1

## Results

This study analyzed data through structural equation modeling (SEM), and for this purpose, partial least square (PLS) SEM was used instead of covariance-based techniques such as AMOS (Nawaz et al., 2022). The PLS-SEM is selected because it is suitable for both studies, that is, confirmatory and exploratory (Avotra et al., 2021). Structural equation modeling consists of covariance-based (CB-SEM) and PLS-SEM techniques (Yingfei et al., 2021). Both approaches have differences, such as the covariance-based is used to acknowledge/refuse the theories. On the contrary, PLS-SEM offers extensions and advances in theories (Hair et al., 2016). Smart PLS 3.3 was used to measure the data. It measured data in two parts: measurement and structural path. Smart PLS is appropriate for complex or even small sample size data analysis.

Reliability and validity of the model are a part of measurement models. This study examined the model's reliability with Cronbach alpha, roh-A, composite reliability, and average variance extract and the model's validity with convergent and discriminant validity (Hair et al., 2014, 2016). This study models all variables' reliabilities and are shown in Table 2. First, according to the threshold of Cronbach alpha, it should be >0.70 (Hair et al., 2019). This study model variable Cronbach alpha values are larger than 0.70. For instance, the values of IV (organizational brand building), DVs (employee-based brand equity and organizational brand equity), and moderators (brand-oriented leadership) are 0.858, 0.884, 0.883, and 0.876, respectively. These values are according to the given Cronbach alpha threshold. Thus, all values are accepted. Second, the roh-A values of all variables are according to the threshold. Third, model variables' composite reliability (CR) and average variance extract (AVE) are also examined. The acceptable values of variables for composite reliability are also >0.7, and the average variance extract is >0.5. This study model variable values are within the limit of the threshold.

**Table 2:** Reliability and validity of the study constructs.

**Construct**	**Item**	**Outer loadings**	**VIF**	**Alpha**	**roh-A**	**Composite reliability**	**AVE**
OBBS	OBBS2	0.892	2.944	0.858	0.869	0.904	0.704
	OBBS3	0.878	2.799				
	OBBS4	0.827	2.011				
	OBBS5	0.751	1.589				
EBBE	EBBE1	0.856	2.306	0.884	0.893	0.92	0.742
	EBBE2	0.909	3.149				
	EBBE4	0.805	2.010				
	EBBE5	0.873	2.613				
OBE	OBE2	0.799	2.715	0.883	0.891	0.91	0.629
	OBE3	0.787	2.456				
	OBE4	0.796	2.674				
	OBE5	0.789	1.895				
	OBE6	0.828	2.463				
	OBE7	0.758	2.049				
BOL	BOL1	0.811	1.883	0.876	0.883	0.909	0.666
	BOL2	0.835	2.463				
	BOL3	0.832	2.454				
	BOL4	0.803	2.748				
	BOL5	0.800	2.650				

*OBBS, organizational brand-building strategies; EBBE, employee-based brand building; OBE, organizational brand equity; BOL, brand-oriented leader*.

**Figure 2:**
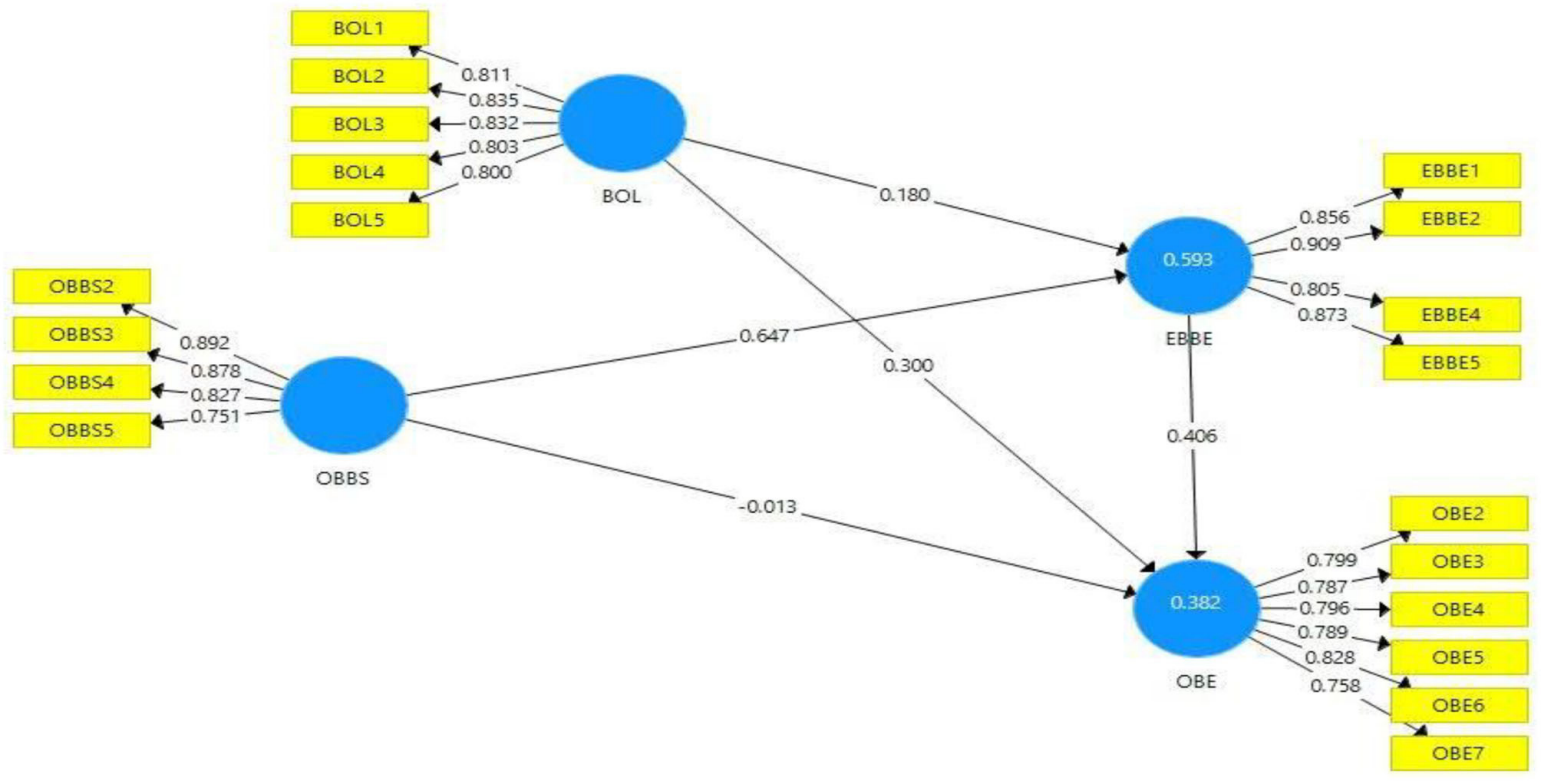
Path estimates.

Moreover, all variables' outer loadings were also checked and are presented in Table 2. A value >0.6 is considered appropriate for items' outer loadings (Figure 2). All variables items are larger than 0.6 instead three items and thus removed; for instance, one item of IV (organizational brand-building strategies) OBBS1 was removed, and two items of DVs (employee-based brand equity) EBBE3 and (organizational-based equity) OBE1 were removed. These items were removed due to weaker or poor loadings and for the better output of the results.

This study also assessed the collinearity issue through the variance inflation factor (VIF). Hair et al. (2014) acknowledged that VIF values below 0.5 are considered acceptable. Table 2 shows that the VIF values of this study model inner constructs are between the range of 1.589 and 2.944. It shows that all items' VIF values are according to the threshold. Thus, no collinearity issue was identified in this study research model.

The *R*^2^ value above 0.25 and up to 0.5 shows a moderate model strength in primary data. This study models DVs such as employee-based brand equity (*R*^2^ = 0.593) and organizational brand equity (*R*^2^ = 0.382) *R*^2^ values showed moderate strength in the model (Hair et al., 2016). Moreover, the *Q*^2^ values of all models' latent constructs are higher than the zero. It is also a sign of significance model.

The discriminant validity of this study was assessed through the Fornell–Larcker criterion and heterotrait–monotrait (HTMT) (Xiaolong et al., 2021). The Fornell–Larcker criterion confirms discriminant validity by taking the square root of all model variables' average variance extract values (Fornell and Larcker, 2016; Hair et al., 2016). Table 3 presents all variables' discriminant validity based on the Fornell–Larcker criterion. This table shows that the model discriminant validity is achieved as the first values of all variables in each column show the highest values compared to their below values (Fornell and Larcker, 2016; Hair et al., 2016).

**Table 3:** Discriminant validity (Fornell–Larcker-1981 Criteria).

**Constructs**	**BOL**	**EBBE**	**OBBS**	**OBE**
BOL	**0.816**			
EBBE	0.573	**0.862**		
OBBS	0.606	0.757	**0.839**	
OBE	0.525	0.568	0.477	**0.793**

*OBBS, organizational brand-building strategies; EBBE, employee-based brand building; OBE, organizational brand equity; BOL, brand-oriented leader*.

According to the HTMT ration criterion, all variables' values <0.85 are considered appropriate. However, HTMT values up to 0.90 are also acceptable (Hair et al., 2016). Table 4 shows that all the values of this study model are according to the given standard as below 0.85 and up to 0.90. These outcomes revealed that discriminant validity is achieved in this study model.

**Table 4:** Discriminant validity (HTMT).

**Constructs**	**BOL**	**EBBE**	**OBBS**	**OBE**
BOL	**–**	**–**	**–**	**–**
EBBE	0.640	**–**	**–**	**–**
OBBS	0.693	0.854	**–**	**–**
OBE	0.586	0.622	0.546	**–**

*OBBS, organizational brand-building strategies; EBBE, employee-based brand building; OBE, organizational brand equity; BOL, brand-oriented leader*.

### Hypotheses Testing

This study applied a bootstrapping method with 5,000 samples for statistical verification of the model hypotheses (Hair et al., 2014, 2016). This study considered *t* and *p* values to reject and accept the hypotheses (Hair et al., 2014). Table 5 explains the outcomes of the H1 relationship that proposed the positive impact of organizational brand-building strategies on employee-based brand equity, and *t* and *p* values confirm this proposition's acceptability (*t* = 10.825, *P* = 0.000). Thus, the H1 is accepted. Moreover, the beta value of this hypothesis showed that one unit change in organizational brand-building strategies would bring a 0.598 change in employee-based brand equity. Second, H2 predicted the positive impact of organizational brand-building strategies on employee-based brand equity. The *t* and *p* values (*t* = 0.028, *p* = 0.978) of H2 showed insignificant outcomes. Hence, H2 is rejected. It confirmed that organizational brand-building strategies do not positively influence the employee-based brand equity. Third, the H3 proposed that the employee-based brand equity positively impacted on organizational brand equity and Table 6 shows significant outcomes as *t* and *p* values showed acceptability (*t* = 5.319, *p* = 0.000). Thus, H3 is accepted. The beta value of H3 revealed that one unit change in employee-based brand equity would bring 0.409 unit changes in organizational-based equity. H5 proposed the positive influence of brand-oriented leadership on employee-based brand equity. The acceptable range for this proposition's *t* and *p* values is (*t* = 3.799, *p* = 0.000). Thus, H5 is accepted. The beta value showed that one unit change in brand-oriented leadership would hold 0.171 employee-based brand equity changes. H6 also proposed the direct impact of brand-oriented leadership on organizational equity. The *t* and *p* values of H6 showed significant outcomes (*t* = 4.510, *p* = 0.000). Hence, H6 is also accepted. According to the path coefficient value of H6, the one unit change in brand-oriented leadership will lead to 0.303 unit changes in organizational brand equity.

**Table 5:** Hypotheses testing.

	**Coefficient (Beta)**	**S.D**	***t***	***p***	**Status**	
**Hypotheses**						
H1	OBBS **→** EBBE	0.598	0.055	10.825	0.000	Supported
H2	OBBS **→** OBE	0.002	0.065	0.028	0.978	Not Supported
H3	EBBE **→** OBE	0.409	0.077	5.319	0.000	Supported
H5	BOL **→** EBBE	0.171	0.045	3.799	0.000	Supported
H6	BOL **→** OBE	0.303	0.067	4.510	0.000	Supported
**Mediation Hypotheses**						
H4	OBBS **→** EBBE **→** OBE	0.245	0.052	4.680	0.000	Supported
**Moderation Hypotheses**						
H7	OBBS * BOL **→** EBBS	−0.055	0.028	1.992	0.046	Supported
H8	OBBS * BOL **→** OBS	0.019	0.044	0.426	0.670	Not Supported

*OBBS, organizational brand-building strategies; EBBE, employee-based brand building; OBE, organizational brand equity; BOL, brand-oriented leader*.

**Table 6:** Direct, indirect, and total path estimates.

	**Beta**	**SD**	***t***	***p***
**Direct path**				
OBBS → EBBE	0.598	0.055	10.825	**0.000**
OBBS → OBE	0.002	0.065	0.028	**0.978**
EBBE → OBE	0.409	0.077	5.319	**0.000**
BOL → EBBE	0.171	0.045	3.799	**0.000**
BOL → OBE	0.303	0.067	4.510	**0.000**
**Indirect path**				
OBBS → EBBE → OBE	0.245	0.052	4.680	**0.000**
BOL → EBBE → OBE	0.070	0.022	3.238	**0.001**
**Total path**				
OBBS → EBBE	0.598	0.055	10.825	**0.000**
OBBS → OBE	0.247	0.063	3.926	**0.000**
BOL → EBBE	0.171	0.045	3.799	**0.000**
BOL → OBE	0.373	0.062	6.023	**0.000**

*OBBS, organizational brand-building strategies; EBBE, employee-based brand building; OBE, organizational brand equity; BOL, brand-oriented leader*.

Table 5 also shows the positive outcomes of the indirect proposition of H4 (*t* = 4.680, *p* = 0.000). As predicted, organizational brand-building strategies positively influence the employee-based brand-building equity, and in turn, employee-based brand building positively influences the organizational brand equity. It shows that employee-based brand equity positively mediates the relationship between organizational brand-building strategies and organizational brand equity. Thus, H4 is accepted.

Moreover, H7 predicted the moderation effect of brand-oriented leadership between organizational brand-building strategies and employee-based brand equity. According to the *t* and *p* values of H7 (*t* = 1.992, *p* = 0.046), brand-oriented leadership has negatively moderated the relationship between brand-oriented leadership and employee-based brand equity. It shows that brand-oriented leadership weakens the relationship between organizational brand-building strategies and employee-based brand equity. Thus, H7 is accepted. Similarly, H8 also predicted a moderation effect of brand-oriented leadership between the association of organizational-based brand strategies and organizational brand equity but *t* and *p* values (*t* = 0.426, *p* = 0.670) showed insignificant outcomes. It shows that brand-oriented leadership did not moderate the relationship between organizational brand-building strategies and organizational brand equity. Thus, H8 is rejected.

## Discussion

Branding is considered an essential tool to compete in today's dynamic market (Huang and Tsai, 2013). In this turbulent environment, organizations put their efforts into finding ways to build brand equity. In this regard, organizational strategies play a key role in the brand-building process of firms (Ayrom and Tumer, 2021). In addition, scholars also acknowledged that employees also have a considerable role in the building process of brand equity (Boukis and Christodoulides, 2020; Liu et al., 2020). With the support of signaling theory and theory of motivation, this study attempts to check the role of organizational strategies in the creation of organizational equity as well as EBBE. Further, this study is also trying to determine the moderating role of brand-oriented leadership between organizational brand-building strategies and organizational equity and EBBE, respectively. This study revealed that organizational brand-building strategies are significant signals for EBBE and organizational brand equity. This study found a positive direct relationship between organizational brand-building strategies and employee-based brand-building strategies such as brand-building strategies enhance firms' values and reputation, which further enhance employees' trust, commitment, and brand credibility (Glynn, 2012). This study did not find a positive direct relationship between organizational brand-building strategies and organizational brand equity. The negative results showed that organizations might not apply suitable strategies for market positioning or maybe they have poor decision-making regarding brand-building strategies (Thed and Fadzill, 2018). This study also examined the mediating role of employee-based brand equity between organizational brand-building strategies and organizational brand equity. The outcomes of this study revealed that employee-based brand equity positively mediated this relationship (Table 5). The outcomes suggest that organizational brand-building strategies may not directly influence organizational brand equity but can influence through the mediation of employee-based brand equity. Thus, organizations need to focus on building employee-based brand equity. Through building internal and external endeavor processes, brand-oriented companies can develop healthy brand awareness, reputation, and trustworthiness with organizational brand among employees (Huang and Tsai, 2013).

Moreover, this study also checked the direct effect of brand-oriented leadership on employee-based brand equity. The outcomes show that brand-oriented leadership positively influences employee-based brand equity. Second, this study examined brand-oriented leadership's direct effect on organizational brand equity. The results revealed that brand-oriented leadership positively influences organizational brand equity. Hence, through brand-oriented leadership, organizations can apply internal brand management and brand communications that enhance employee brand commitment and engagement (Afshardoost et al., 2021).

Furthermore, this study also assessed brand-oriented leadership as a moderator in the relationship between organizational brand-building strategies and employee-based brand equity. The outcomes showed that brand-oriented leadership reduces the strength of organizational brand-building strategies and employee-based brand equity relationships. Second, this study assessed the moderator role of brand-oriented leadership in the relationship between organizational brand-building strategies and organizational brand equity. This study results showed that brand-oriented leadership does not moderate the relationship between organizational brand-building strategies and organizational brand equity.

### Discussion on Unexpected Moderating Results

The moderating effects of brand-oriented leadership on the relationship between organizational brand-building strategies and employee-based brand equity are unexpected. Brand-oriented leadership should positively impact as a moderator between organizational brand-oriented leadership and employee-based brand equity (Maleki Minbashrazgah et al., 2021). Second, it is also unexpected that brand-oriented leadership does not moderate the relationship between organizational brand-building strategies and organizational brand equity. The author tried to know the consequences of brand-oriented leadership's unexpected moderation results. The author interviewed brand-oriented leaders. The outcomes of the interviews revealed that most of the brand-oriented leaders were not involved in building organizational brand strategies. The organization's upper-level management prepared the strategies without consulting their brand-oriented leaders. In this regard, the organizations did not utilize the capabilities of their leaders, and as a consequence, leaders were not happy with the organizational brand-building strategies. Hence, it was the prime cause of contradictions between organizational brand-building strategies and brand-oriented leaders. Thus, organizations should make rational decisions and wise policies to resolve these contradictions for smoothness in building organizational brand equity (Zhao et al., 2019).

## Theoretical and Practical Implications

This study has many theoretical and practical implications. First, theoretically, this study extends the literature on organizational brand-building strategies. Previous studies did not consider organizational brand-building strategies to build employee and organizational brand equity (Järventie-Thesleff et al., 2011; Quaratino and Mazzei, 2018). With the support of signaling theory and theory of motivation, this study took the initiative and extended the literature on the role of organizational brand-building strategies in building brand equity from the employee and organizational perspective. Second, this study examined the mediating role of employee-based brand equity between organizational brand-building strategies and organizational-based brand equity. Hence, this study extended the literature on employee-based brand equity by checking its role as a mediator. Third, this study also extended the literature on brand-oriented leadership as brand-oriented leadership positively influences employee-based brand equity and organizational brand equity. This study also extends the literature on brand-oriented leadership as a moderator between organizational brand-building strategies and employee-based brand equity. Practically, this study guides the managers that brand-oriented organizations must have differentiation product capabilities that strategically align with organizational brand goals to build employee-based brand equity and ultimately result in organizational brand equity (Huang and Tsai, 2013).

Moreover, brand-oriented leadership plays a crucial role in positively impacting employee-based brand equity and organizational brand equity. This study suggests that top management must coordinate with brand-oriented leadership while building organizational brand strategies for smooth functioning in organizations.

## Limitation of the Study

This study serves the literature in many different ways, but this study also has some limitations. First, this study collected data from different employees working in beverage companies in China in three waves. Future research can conduct a longitudinal study by enlarging the sample size to verify our study results. Second, future research may collect data from other industries by adopting this study model to strengthen the results of this study. Third, this study examined brand-oriented leadership as moderating construct between organizational brand-building strategies and employee-based and organizational brand equity. Future research may consider other moderators like the organizational culture. This study did not check the mediating effect between organizational brand-building strategies and employee-based brand equity relationships. Future research can also propose this relationship with the support of mediators. Moreover, this examined brand-oriented leadership's direct effect on employee-based brand equity and organizational brand equity and found positive outcomes. Future research can confirm this relationship with the support of mediators and moderators. Finally, this study was conducted in China. A similar study could be conducted in other developing countries or developed countries to strengthen and ensure the results.

## Conclusion

In this era of competition, organizations are now well aware of brand equity importance from the organizational and employees' perspectives. Firms are serving their efforts and resources to maintain their sustainability by creating brand equity. This study attempts to examine the role of organizational brand-building strategies on employee-based brand equity and organizational brand equity. The role of leadership in creating brand equity is also worthwhile because frontline employees are considered in the literature as ambassadors of the brand. This study also considers the role of brand-oriented leadership in the theoretical model for empirical investigation. This study's findings depict that organizational brand-building strategies positively influence employee-based brand equity, but their significant influence on organizational brand equity was not found. This study also examined the mediating role of employee-based brand equity in the relationship between organizational brand-building strategies and organizational brand equity and found that employee-based brand equity positively mediates this relationship. This study also assessed brand-oriented leadership's direct relationship with employee-based brand equity and organizational brand equity and discovered its positive effects. Moreover, this study also evaluated brand-oriented leadership as a moderator between organizational brand-building strategies and employee-based brand equity and found that brand-oriented leadership negatively moderates this relationship. Second, this study also considered brand-oriented leadership as a moderator between the association of organizational brand-building strategies and organizational brand equity; however, outcomes revealed that brand-oriented leadership does not moderate this relationship.

## Data Availability Statement

The original contributions presented in the study are included in the article/supplementary material, further inquiries can be directed to the corresponding author/s.

## Ethics Statement

The studies involving human participants were reviewed and approved by Tianjin Vocational Institute, China. The patients/participants provided their written informed consent to participate in this study. The study was conducted in accordance with the Declaration of Helsinki.

## Author Contributions

ZW has conceptualized the concept, collected data, and wrote the draft.

## Conflict of Interest

The author declares that the research was conducted in the absence of any commercial or financial relationships that could be construed as a potential conflict of interest.

## Publisher's Note

All claims expressed in this article are solely those of the authors and do not necessarily represent those of their affiliated organizations, or those of the publisher, the editors and the reviewers. Any product that may be evaluated in this article, or claim that may be made by its manufacturer, is not guaranteed or endorsed by the publisher.
